# Landscape Management of Fire and Grazing Regimes Alters the Fine-Scale Habitat Utilisation by Feral Cats

**DOI:** 10.1371/journal.pone.0109097

**Published:** 2014-10-15

**Authors:** Hugh W. McGregor, Sarah Legge, Menna E. Jones, Christopher N. Johnson

**Affiliations:** 1 School of Biological Sciences, University of Tasmania, Hobart, Tasmania, Australia; 2 Australian Wildlife Conservancy, Derby, Western Australia, Australia; 3 Australian Wildlife Conservancy, Mornington Wildlife Sanctuary, Derby, Western Australia, Australia; University of New South Wales, Australia

## Abstract

Intensification of fires and grazing by large herbivores has caused population declines in small vertebrates in many ecosystems worldwide. Impacts are rarely direct, and usually appear driven via indirect pathways, such as changes to predator-prey dynamics. Fire events and grazing may improve habitat and/or hunting success for the predators of small mammals, however, such impacts have not been documented. To test for such an interaction, we investigated fine-scale habitat selection by feral cats in relation to fire, grazing and small-mammal abundance. Our study was conducted in north-western Australia, where small mammal populations are sensitive to changes in fire and grazing management. We deployed GPS collars on 32 cats in landscapes with contrasting fire and grazing treatments. Fine-scale habitat selection was determined using discrete choice modelling of cat movements. We found that cats selected areas with open grass cover, including heavily-grazed areas. They strongly selected for areas recently burnt by intense fires, but only in habitats that typically support high abundance of small mammals. Intense fires and grazing by introduced herbivores created conditions that are favoured by cats, probably because their hunting success is improved. This mechanism could explain why, in northern Australia, impacts of feral cats on small mammals might have increased. Our results suggest the impact of feral cats could be reduced in most ecosystems by maximising grass cover, minimising the incidence of intense fires, and reducing grazing by large herbivores.

## Introduction

Predator-prey relationships are strongly influenced by the structure and quality of habitat, principally its vegetation [1], [2], [3]. Variability in vegetation structure may be used by predators to increase hunting success. For example, lions use dense vegetation to hide their approach from prey [4]. Also, it may be used by prey to help them evade predators, such as elk using woodlands as a refuge from wolves [5]. Changes in habitat structure may therefore shift the relationships between predators and prey [6], [7]. Such changes can determine the extent to which some prey are threatened with extinction by heavy predation [2], [8].

One of most pervasive impacts on vegetation structure arises from changes to fire and grazing regimes. While drivers of such changes vary immensely, the impacts on fauna communities display some general trends. For example, small mammal populations are especially sensitive, and the vast majority of studies detecting declines in populations in response to either intense fire events or intense grazing [9], [10], [11], [12], [13], unless they occur in ecosystems with few predators [14], [15]. The underlying mechanisms of these declines remain elusive, but are likely to be indirect rather than through direct effects such as being burnt by the fires or trampled by cattle [16]. Instead, such disturbances may improve habitat for predators in ways that increases their impacts on prey [9], [17], [18], although no field data are available to confirm this.

Many small mammal species are declining in the savannas of northern Australia, and several are threatened with extinction [18], [19]. Declines have been greatest in areas subject to intense fires [20], [21] and recent experimental evidence also supports an association of grazing by introduced herbivores (cattle, horses, donkeys, buffalo) with the magnitude of small-mammal decline [22]. Both fire and grazing regimes in northern Australia have intensified substantially over recent decades in ways that could contribute to the contemporary native mammal decline. These changes to fire and grazing have generally made grass communities less complex and more open [23].

Predation by feral cats *Felis catus* may also be contributing to the declines. This is suggested by three lines of evidence. First, the declining species fall within the preferred prey-size range of cats [18], [24]. Second, mammal populations in complex rocky habitats have been less affected than those in more productive woodlands and savanna, suggesting a predation effect [25], [26]. Finally, populations of declining mammal species are more stable in the absence of cats, on island or in large enclosures [27], [28]. However, there is a temporal mismatch between the arrival of cats in northern Australia up to 170 years ago [29], and mammal declines observed in the last 20 years [19].

The apparent mismatch in timing of the early arrival of cats and recent mammal declines could be explained by the hypothesis that cat predation has its largest impact when it interacts with fire and grazing regimes established in the more recent past. Reduction of structural complexity of vegetation and increased openness due to fire and grazing might increase the exposure of small mammals to predators, making prey easier to detect and capture [15]. Small mammals are cats' preferred prey [24], [30]. If cats preferentially use the open and relatively simple habitats created by fire and grazing, the result could be a higher predation impacts on small mammals. This has been suggested as a possible mechanism for these declines [19], [31], however, until now there has been no evidence.

If cats do favour the conditions created by fire and grazing, this should be revealed by the patterns of movement of individual cats in heterogeneous landscapes with variable effects of fire and grazing. We tested this hypothesis using intensive GPS tracking of a large sample of individual cats; both within and outside of a large 40 300 hectare area that has been destocked of all introduced herbivores (cattle, horses, and donkeys) [22], and spanning contrasting fire patterns (mild control fires or intensive wildfires). A dynamic habitat map was created so that fire and vegetation attributes at any location or point in time could be determined. This was used to generate a parsimonious model of fine-scale habitat selection by cats. We predicted that feral cats would select for open grass cover to improve hunting success. If so, we hypothesise that cats would increase their use of habitats that have been recently burnt or intensely grazed, and that this relationship would be stronger in areas of high small-mammal abundance.

## Methods

### Study area

**Figure 1:**
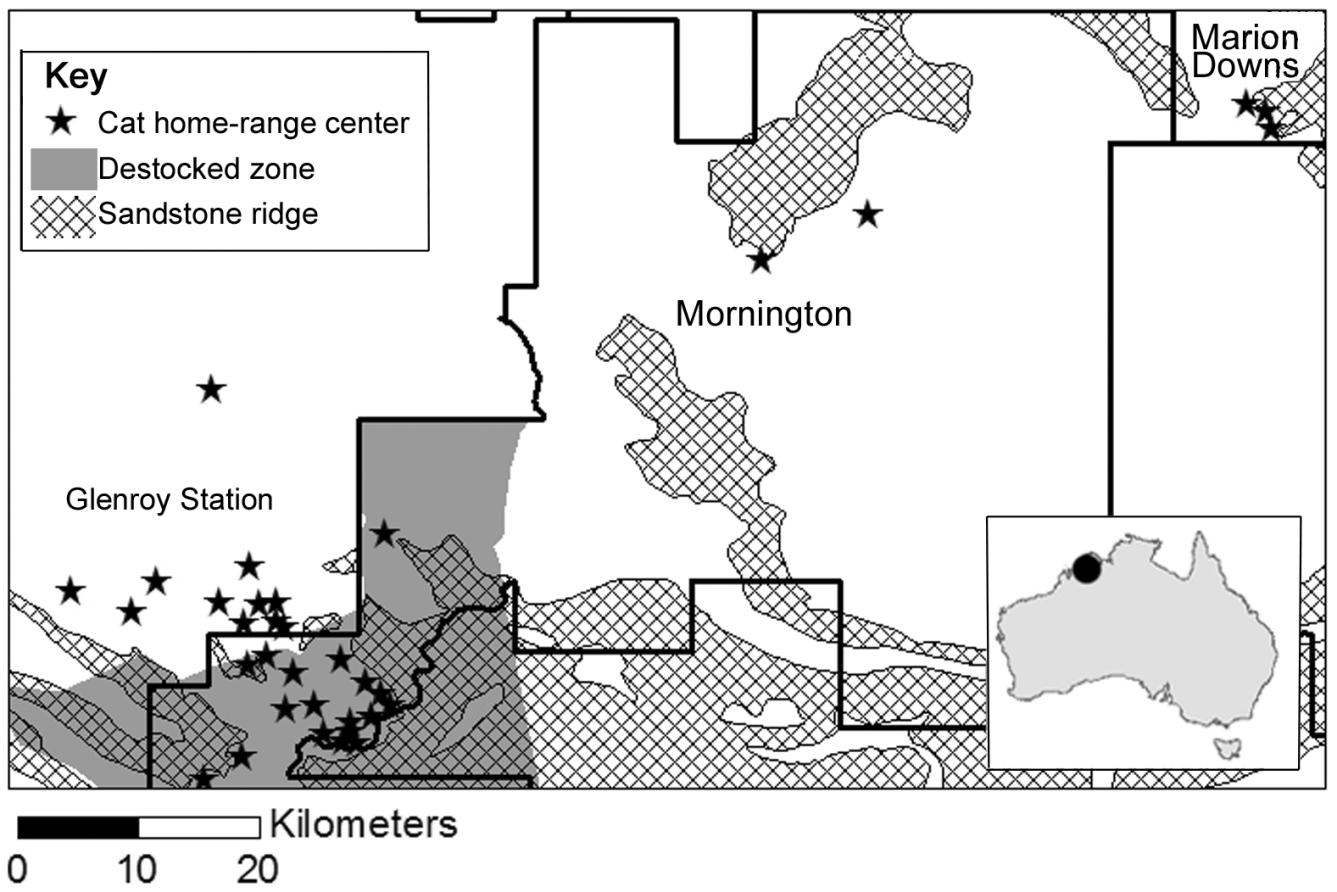
Map of study area in north-west Australia (see inset), including home-range centroids of feral cats used in this study. The dark grey represents the destocked zone.

Our study area encompassed three large properties in the central Kimberley of north-western Australia (**17°01′S, 126°01′E**, see Fig. 1). One property is managed for commercial cattle production (Glenroy, 1455 km^2^) and two are ex-pastoral leases managed for conservation by the Australian Wildlife Conservatory (Mornington and Marion Downs Sanctuaries, 3225 km^2^ and 2676 km^2^ respectively). Habitats are mostly savanna woodlands with a perennial grass layer, dissected by riparian vegetation along the edges of creeks. The region has a tropical monsoon climate with three broad seasons: the wet (December – March), early dry (April – July) and late dry (August – November). Fire is managed on all three properties to promote biodiversity values. This fire management aims to reduce the incidence of extensive, high-intensity, uncontrolled fires in the late dry season using strategic prescribed burning in the early dry season when fires tend to be small and of low intensity because of weather and condition of the grass layer. In addition, when uncontrolled late dry season fires occur, they are suppressed where possible. All large introduced herbivores (cattle, horses and donkeys) have been removed from a 40,300 ha fenced section of Mornington since 2005 [22], - hereafter referred to as the ‘destocked’ zone. The only other large mammalian predator in the study area is the dingo *Canis dingo*. Whilst they are controlled elsewhere in Australia, they are not persecuted in the study area, and occurred at a density of ∼0.2 per km^2^ [32].

### Cat capture and tracking

Feral cats were captured between September 2010 and June 2013, using either large wire cage traps, leg-hold traps (soft-jaw, size #1.5) or by spotlighting and netting with the assistance of dogs trained to locate and bail cats up trees. If a cat was either bailed up a tree or required examination of possible injury, it was sedated with Zolotil at a rate of 0.5 cc/kg via intramuscular injection. Cats were fitted with GPS collars (Telemetry Solutions Quantum 4000 enhanced). Cats weighing between 2 and 3.3 kg were fitted with a 70 g collar, and those weighing more than 3.3 kg were fitted with a 100 g collar (<3% of body-weight). Sedated cats were released after full muscle control was regained (4–6 hours later), and non-sedated cats were released as soon as possible (2–5 minutes later). When it was necessary to replace GPS collars, the cats were recaptured using the dogs.

GPS collars were deployed on equal numbers of cats in the stocked and destocked zone, and between burnt and unburnt areas. Within burnt areas, the cats were split evenly between areas with low and high intensity fires (Table 1). The GPS units were programmed to record fixes every 15 minutes for two-day bouts, starting and finishing at 12 pm. These bouts were separated by intervals of one, two or fourteen days. All bouts were timed to commence at least 24 hrs after the cat was handled. Units were programmed to search for a satellite for 60 seconds, and to remain on for at least 5 seconds to refine the fix if there was memory from the last fix, or 15 seconds if not.

**Table 1:** The number of cats fitted with GPS collars in each of the different grazing and fire treatments. Destocked means that all introduced herbivores are excluded.

Management	No fire	Mild fire	Intense fire	Total
Stocked	8 (5**♂** 3**♀)**	4 (3**♂** 1**♀)**	4 (3**♂** 1**♀)**	**16**
Destocked	8 (6**♂** 2**♀)**	4 (4**♂)**	4 (4**♂)**	**16**
**Total**	**16**	**8**	**8**	**32**

### Habitat variables

Across the study area, we developed habitat maps relating to fire and grazing, along with any other variable likely to influence cat habitat selection or movement. Where necessary, the maps were updated to make them temporally dynamic, so that attributes at any given time and location could be determined. Most descriptors of habitat related to the ground layer, rather than the tree layer. Nine distinct grass communities common in the region (see Table 2) were mapped by examining colour disjunctions on aerial photos while altering light levels in Photoshop Elements v. 8, tracing the boundaries of discrete polygons onto the aerial photos, then geo-rectifying these boundaries in ArcGIS v.10. For example, spinifex *Triodia* spp. grasslands are uniquely green in dry-season aerial photos, while communities dominated by bluegrass *Dichanthium fecundum* are white. The digital map was ground-truthed at 768 plots (described later); the attribution of grass community was correct at 96% of sites.

**Table 2:** Vegetation communities mapped in the study region.

			Small mammal abundance					
		Grazing rank	Stocked			Destocked		
Grass community	Dominant grasses		2011	2012	2013	2011	2012	2013
Riparian forest^A^	*Chrysopogon fallax*	1	0.2	0.3	0.8	1.2	3.4	9.4
	*Mnesithea rottboellioides*							
	Various introduced grasses							
Alluvial grasslands^A^	*Crysopogon fallax*	2	0.2	0.3	0.8	1.2	3.4	9.4
	*Dichanthium fecundum*							
Bluegrass plains	*Aristida* spp.	3	1	1.3	4.6	0.3	2.6	2.9
	*Dichanthium fecundum*							
Canegrass^A^	*Mnesithea rottboellioides*	4	0.2	0.3	0.8	1.2	3.4	9.4
Mixed woodlands	*Aristida* spp.	5	0	1.3	0.6	0.2	3.8	6
	*Dichanthium fecundum*							
	*Heteropogon contortus*							
Sandseep	*Triodia* spp.	6	1.7	1.2	1.2	1.8	7.4	16.8
Hillside woodlands^B^	*Sehima nervosum*	7	0	0.6	1	0.4	3.2	4
Spinifex woodlands^B^	*Triodia* spp.	8	0	0.6	1	0.4	3.2	4
Bare ground	No or little	9	0	0	0	0.2	0.2	0.5

^A^ and ^B^ denote grass communities where the small mammal abundance was grouped, as sites were typically larger than the mapped distributions of these communities.

Vegetation classes are ranked by their preference for grazing by domestic stock, with 9 the most impacted and 1 the least (Grazing rank). Small mammal abundance is number of small mammals captured per 100 trap nights in the stocked and destocked zone, from 2011 to 2013.

Fire scars were initially mapped using monthly Landsat 7 remote-sensing imagery available from the US Geological Survey (2011–2013), and fire boundaries were then refined using aerial photography taken from a helicopter flying approximately 300 m above ground. For each burnt area, we assigned the date of burn and intensity (intense = 100% tree scorch and no ground cover remaining unburnt, or mild  =  all other fires). Relative to the date of each GPS fix, fire was considered in multiple binary variables at 30, 60, 90, 180, 360 or 600 days since fire. The Australian Wildlife Conservancy's stock-proof fence [22] separated the stocked and destocked areas (see Fig. 1).

A dynamic map was created that estimated grass cover at any given location and time since fire, based on a series of models of the response of the grass layer to fire (given grass community, stocking status). They were created from field data, where vegetation attributes were measured at 768 plots (each 10 m^2^) across the study area and duration, spread equally across grass communities (see Table 1) and combinations of mild/intense fire, time since fire, and grazed and destocked areas (total of 96 plots per community). At each plot, we estimated the extent of grass cover at different heights by adapting a line-intercept method. We inserted a 100 cm pole (diameter of 1.5 cm) vertically through the grass to the ground at 50 points in a systematic grid over the plot. The number of grass intercepts was recorded in height intervals of 0–10 cm, 11–30 cm, and 31–100 cm. This was used to derive grass cover, cover of dense tussocks, and relative biomass at each plot (see Material S1 for more detail on methods). These variables were modelled against grass community, time since last fire, intensity of fire, and stocking status. Models were then used to derive values for all GPS fixes and random points used in discrete choice models (see Material S1).

The influence of grazing on grass biomass was measured as the difference in the average total number of grass intercepts per plot between grass communities in the stocked and destocked sites. We used only unburnt sites for this comparison. These averages were converted into a grazing impact rank. However, these variables would be confounded by correlation as the habitats favourable to cattle would also be favoured by feral cats (e.g. riparian areas), irrespective of impacts of cattle. Therefore, this score or rank was applied to all fixes, grazed or not, and the actual impacts of cattle grazing considered as the difference in this variable's strength between the destocked zone and outside.

Relative small-mammal abundances in different habitats were estimated from the Australian Wildlife Conservancy's annual fauna monitoring data, which is carried out across all three properties [32]. The sample at each site is based on 60 trap-nights using small mammal box traps (Elliott traps) and 12 nights of possum-sized wire traps, with 55 to 64 sites sampled each year - totalling 176 site-years between 2011–3. Small mammals were defined as those weighing between 30 g and 2000 g [18], [24], and were predominantly *Rattus tunneyi* and *Pseudomys nanus* (these species comprised 87% of captures). For each year, the average rates of capture of individual small mammals were calculated for each grass community in the grazed and destocked areas (see Table 2). However, as sites were typically larger than the mapped patterns of these grass communities, results from some communities were combined (see Table 2). In the event cats altered their response to a threshold of small mammal abundance, we also included three binary variables of abundance (whether there was more than 1, 2 or 5 small mammal captures per plot) for each habitat.

We measured other spatial and temporal features likely to be important to cats to provide context for their movements. As cats may prefer hunting on edges between open and dense vegetation, we delineated a 25 m buffer either side of any linear boundary where grass cover was <25% on one side and >50% on the other (these were most often fire edges). Water features were mapped in the field, and each assigned a descriptor for the seasons that they contained water (wet season only, wet and early dry, or year-round). This was used to derive distance to known standing water at any given time throughout the study. Elevation and slope were derived from a digital elevation model of 15 m resolution. We created a variable representing the spatial home-range context for each cat, by making a kernel density estimate (smooth cross-validation) for all moving fixes of each cat, and delineating kernel isopleths at 50%, 90%, 95% and 99% contours. Temporal variables included sun time [33], season (wet, early dry or late dry), number of months since the end of the wet season, and minimum nightly temperature (HOBO temperature data logger, MicroDAQ). Finally, a cat's choice of which habitat to select in successive GPS fixes may be dependent on the type of habitat it was last in. This spatial correlation might arise when an animal selects for certain landscape features and tends to remain within them for some time, rather than making a *de novo* selection at every 15 min interval of their walk. Therefore, we fitted a binary ‘carry-over’ variable, which described whether the vegetation, grazing and fire values were equal to the previous fix.

### Organisation of data

All GPS fixes that were likely to be erroneous, biased or to represent a stationary cat were removed from the analysis. Erroneous fixes were those representing implausible ‘spikes’ in movement, presumably caused by GPS error [34]. We deemed it unlikely that a cat would suddenly change course and speed, then return to the same area it was in 15 minutes ago, so spikes were defined where fixes met all the following criteria: distances from the last fix were >50 m, difference in distance from the preceding and succeeding fix was <10%, and turning angle >170° [34], [35]. The HDOP values were not used to filter fixes, as a test of six GPS collars found no relationship between HDOP and distance from the GPS fix to the known location. Fixes that were potentially biased by human disruption of the behaviour of the cat were removed, being those within three hours of field VHF tracking (this was occasionally carried out in order to download data remotely from the cat GPS collar). Fast-moving fixes were also removed, as it was likely cats were moving away from something, rather than choosing habitat. For this purpose a filtering speed of greater than 2 km per hour was used, as this was where the histogram of speeds between fixes reached an asymptote [35], representing a shift in behaviour mode. A test of GPS error within the open savanna found that 95% of fixes had <5 m error (from 634 fixes on six different collars), so fixes less than 10 m from the preceding fix were classed as stationary. We considered only moving fixes, as cats may have different habitat requirements for resting versus hunting.

### Data analysis

Habitat selection by cats was analysed using discrete choice modelling [36]. The range of ‘available’ habitats was calculated for each fix, and then we compared the option selected by the cat to the available habitats. To find the available habitats, we first constructed probability distributions of a cat's step-length and bearing over 15-minute intervals, then used these probability distributions to select five random points to sample the cat's options [36]. Each GPS fix and associated random points were attributed with the habitat variables of interest using the dynamic vegetation map.

We determined resource selection by cats by creating models with all combinations of variables, including different biologically relevant interaction terms, and comparing them within an information theory framework [37]. This produced a total of 916 models. For each interaction term a model was included with all combinations, or with only significant combination terms retained. No pairs of variables with Pearson's correlation greater than 0.5 were included in the same model. Models for habitat selection were created using standard case-control logistic regression models, and were implemented in R [38] using the ‘clogit’ command from the ‘survival’ library. Each individual cat was considered as a mixed effect in the models, using Gaussian frailty [39]. The most parsimonious models of cat habitat selection were chosen as those with an AIC score within two points of the highest-ranked model [37], and only these are presented in the results. The cat's selection is measured as an odds ratio representing the magnitude of change in the odds of selection for each unit of the predictor variable. Differences in the odds ratio are relative only to the other habitat choices immediately available to a cat.

### Ethics statement

All data collection fulfilled all legal requirements in Australia, and has been approved by University of Tasmania Animal Ethics Committee (A0011661) and Western Australian Department of Parks and Wildlife Animal Ethics Committee (2010/35), with a Regulation 17 licence to research animals (SF009379). All research was conducted with permission on three pastoral leases; Mornington Wildlife Sanctuary, Marion Downs (both managed by the Australian Wildlife Conservancy, ph; +61 8 9191 7014), and Glenroy Station (ph: +61 8 9191 4703). All three leases are located around 17°01′S, 126°01′E. Field studies did not involve protected or endangered species.

## Results

In total, 60 cats were captured between September 2010 and June 2013. Three cats were caught in wire cage traps (265 trap nights), 19 in leg-hold traps (940 trap nights) and 38 by spotlighting/netting with trained cat-dogs (221 hours). GPS collars were placed on 37 cats, and at least one month of GPS data was obtained from 32 cats whose locality was spread equally across stocking and fire management treatments (Table 1). There was a strong male bias in the sample of cats: males comprised 78% of all captured cats (47/60) and 78% of cats from which GPS data were obtained (25/32). Of the GPS-collared cats, four had disappeared and their fates were unknown at the end of the study, nine had died naturally, and the rest were euthanased.

From the 32 cats that provided useable GPS data, we obtained a total of 133 047 GPS fixes. Cats were moving 56% of time. Of these moving fixes, 62% were at night (between sunset and sunrise). Removal of erroneous or biased fixes and those representing high-speed movement left 38 472 choices for habitat between successive 15-minute moving fixes.

From the 916 models generated to describe habitat selection by cats, three were ranked within the candidate model set (the model with the lowest AIC score and two other models within two AIC scores of the top model). The top model carried 41% of the weight, compared to 24% and 20% for the second and third ranked models. Of these, the second and third ranked models were almost identical to the top model, but contained interaction terms that did not decrease the models AIC value. As these terms did not improve the maximum likelihood for these models, only the top model was considered further (Table 3).

**Table 3:** Statistics of the top ranked model of cat habitat selection based on GPS data at 15 minute intervals from 32 individuals.

Variable	Odds ratio	robust SE	Z	Pr(>|z|)	
Grass cover with scarce small mammals^a^	−1.26	0.08	−2.88	0.004	**
Grass cover with abundant small mammals^a^	−1.85	0.1	−6.44	0.0001	***
Bare/grass edge with scarce small mammals^a^	1.2	0.07	2.81	0.005	**
Bare/grass edge with abundant small mammals^a^	1.41	0.06	6.26	<0.0001	***
Fire scar <360 days	−1.32	0.07	−4.09	<0.0001	***
Intense fire scar <360 days old	1.54	0.13	3.29	0.001	**
Intense fire scar <90 days old	−2.11	0.13	−5.67	<0.0001	***
Small mammal abundance (square-root)	3.52	0.21	6.03	<0.0001	***
Small mammal abundance, intense fire scar <360 days	−4.93	0.66	−2.4	0.0163	*
Small mammal abundance, intense fire scar <90 days	10.71	0.79	2.99	0.0028	**
Grass communities ranked on grazing impacts, in:					
- stocked areas during day	5.96	0.14	12.56	<0.0001	***
- destocked areas during day	3.52	0.25	5.07	<0.0001	***
- stocked areas over night	2.64	0.17	5.65	<0.0001	***
- destocked areas over night	2.44	0.16	5.67	<0.0001	***
Water proximity (km) by months into dry season	1.56	0.06	−7.77	<0.0001	***
- as above, by minimum nightly temperature (10°C)	1.13	0.03	3.94	<0.0001	***
Elevation (100m)	−1.93	0.28	−2.32	0.0202	*
Home range isopleth^b^, adult female	3.53	0.05	27.17	<0.0001	***
Home range isopleth^b^, adult male	2.28	0.1	−8.41	<0.0001	***
Home range isopleth^b^, sub-adult	1.06	0.49	−0.12	0.9077	
Same habitat as last fix, if within 95% isopleth	2.06	0.02	41.4	<0.0001	***

a Scarce and abundant small mammals are defined as less than or greater than two individuals captured per 100 trap nights at Australian Wildlife Conservancy monitoring sites.

b Home range isopleth derived at 50, 90, 95 and 99% contours from kernel density estimator.

The odds ratio is the change in selection likelihood per unit of the variable.

The top model included negative selection for grass cover (equivalently, positive selection for open areas) and positive selection for edges (see Table 3). Both variables had a significant interaction with small-mammal density in a binary format (>2 captures per 100 trap nights), showing that cats selected particularly strongly for open areas in habitats with higher density of small mammals.

**Figure 2:**
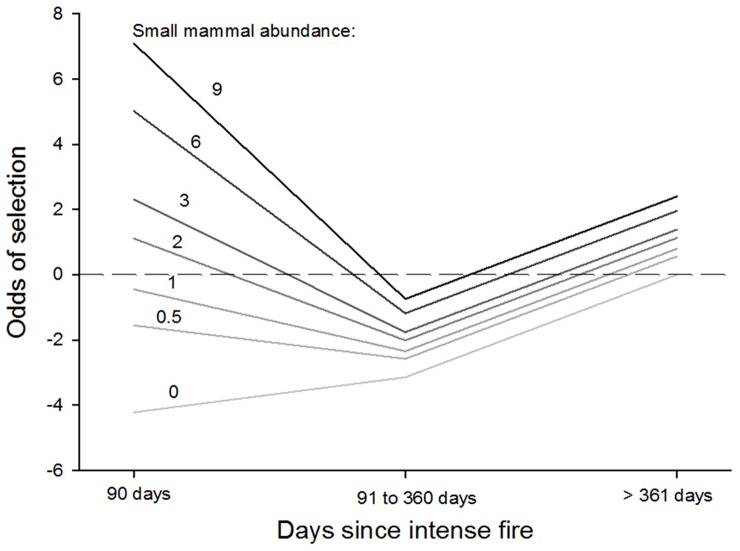
Change in the odds of selection ratios for different days since intense fires, at different average small mammal abundances based on capture rates per 100 trap nights (ranging from 0 to 9, lighter to darker respectively). All other variables in the model are assumed to be constant (see Table 3).

Fire was represented in the top model with fire scars up to 360 days old, fire scars <90 days old, whether the fire was intense, small-mammal abundance, and interaction terms between these variables. Once the odds ratios of these variables were combined, cats showed strong positive selection for areas within 90 days after an intense fire and where density of small-mammal prey was high (Fig. 2). However, in all other circumstances selection for areas burnt by intense or mild fires was negative.

**Figure 3:**
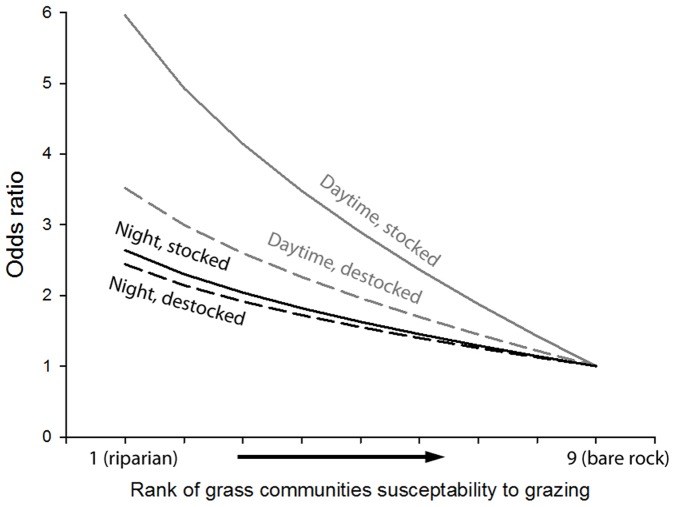
Odds ratios for selection of cats at night (black) and day (grey) in stocked (solid) and destocked (dashed) areas against grass communities ranked by grazing susceptibility. All other variables in model assumed to be constant (see Table 3).

Vegetation types with greater susceptibility to grazing impacts (e.g. riparian areas, Table 2) were strongly selected for in both the grazed and destocked zones. However, this relationship was significantly stronger in the stocked zone than in the destocked zone, especially during the day (see Fig. 3).

Several other variables influenced selection by cats. Cats chose to move towards water. The odds ratio of moving towards water became progressively stronger throughout the dry season (by 0.56 every month into the dry season) and on days with higher minimum temperature (by 0.13 for each 10°C increase). Cats selected against changes in elevation, with odds declining by 0.93 every 100 m. Cats tended to move into higher-use areas within their home range (determined by kernel density estimates), with an interaction with age and sex. Adult females demonstrated the strongest fidelity to home-range isopleths, while for sub-adults of either sex this was not significant (P = 0.9). Cats were twice as likely to select for the same habitats as the previous fix (P<0.001), assuming the fix was inside a cats' home range (95% isopleth).

## Discussion

Our study provides a detailed analysis of the preferences that underlie movement decisions by feral cats in a tropical savanna environment in northern Australia. We show that modifications of habitat produced by grazing and by certain types of fire have strong effects on cat movement behaviour, with the general result that fire and grazing can create habitat conditions which are strongly favoured by cats. When faced with choices about where to move, cats consistently selected sites with a more open grass layer, which had recently been subject to intense fires, and which were heavily grazed. Further, the influence of fire intensity on selection of habitat by cats was strongly affected by whether the habitats in question supported high densities of small mammals: sites in mammal-rich habitats that had recently been burnt at high intensity were especially strongly favoured.

This interaction between cat movements, fire and grazing regimes may help explain the recent declines in small mammals in northern Australia. There has been doubt that predation by cats might be driving these declines because of the mismatch in timing between the introduction of cats and small mammal declines, together with the fact that we have no evidence that small-mammal declines have coincided with an increased populations of cats [19]. As our results demonstrate, impacts of cats could have become more severe with the changes in fire and grazing regimes that began around the 1960s [40], [41], even if cat density remained constant.

Selection by cats for areas with an open grass layer is a consistent finding from other studies on habitat selection by small felids [42], [43], except in situations where moving into open areas leaves them exposed to larger predators [44] or where prey density is low [45]. Our cats' selection for open areas is almost certainly an expression of hunting preferences, as their selection became stronger in areas with higher small-mammal abundances. Although grass cover in itself had a large effect size (odds ratio maximum of 1.8), the effects of grazing and fire patterns on cat movements were much stronger.

Selection for fire scars was strongly dependent on fire intensity and time since fire. In general, cats avoided fire scars up to 360 days after fire. The exception was for recent scars resulting from intense fires in areas where small mammal densities were high, as this created the greatest increase in the odds ratio of any variable in this study. Fire opens up the grass layer thereby leaving prey more exposed and accessible to predators [15]. Intense fires would create pulses where prey would be easy for cats to catch - provided local abundance of prey was high. Cats did not select for recent mild fire scars, even in habitats with high small-mammal abundance. Mild fires typically leave pockets of unburnt vegetation [46], which provide protection for prey [10]. Also, mild fires are often stopped by riparian and alluvial areas [40], and such areas act as refugia for small mammals post-fire [10]. Our results suggest that cats are not able to capitalise on vulnerable small mammals after mild fires, but can after intense fires. This can explain why declines of some threatened native mammals have been so dramatic under regimes of consecutive high intensity fires [20], [21].

Grazing by introduced herbivores also affected habitat choice by cats. Certain vegetation types (with more palatable grasses) were more preferred by introduced herbivores than others - typically riparian areas and bluegrass plains [47]. Cats selected these habitats even when they had been destocked, but grazing intensified this preference. Cats may have a stronger selection for these areas in the grazed zone as lower prey densities [22] require them to hunt in areas of relatively higher mammal density in order to obtain enough food. Daytime movements of cats were especially affected by grazing, suggesting that grazing creates favourable conditions for hunting of diurnal prey, such as quail [48].

Adult cat movements at small-scale 15 minute segments were dictated by their overall home range. Females had a stronger home-range fidelity than males, probably due to the importance of staying near dens that are used for rearing young [49]. Sub-adults displayed no such home range fidelity as they were probably in the process of creating and defining their home-ranges.

The top-order predator, the dingo, would also have influenced the movements of cats. Over the area and duration of the study, the dingo populations was abundant (0.2 individuals per km^2^) and stable [32], and dingoes would have been a constant threat to cats [50]. A concurrent study in the same area that compared GPS movements of cats and dingoes found that although high use areas of dingoes and cats overlapped, cats were constantly avoiding the locations of individual dingoes [51]. This suggests avoidance of dingoes would have affected the timing of behavioural decisions of cats [52], but not necessarily by excluding them from certain areas [53]. Considering dingoes potential role in trophic regulation [25], further research into the relationship between dingoes, cats, and fire and grazing is warranted.

## Conclusions

We demonstrate increased predator activity after intense fires and with grazing by large herbivores, which is likely to increase predation rates on small mammals. Small mammals are the preferred prey of cats, and form a substantial part of the diet of cats in the study region (55% of prey volume, from 33 stomach contents; unpublished data). Furthermore, the preference of cats for open and intensely burned areas depended on small-mammal abundance, and was reduced in habitats where abundance of small mammals was low. These patterns of habitat selection by cats correspond with declines of small mammal populations with intensifying fire and/or grazing regimes in mainland northern Australia, outside complex rocky outcrops [20], [21], [22], [54], [55], [56], [57]. Our work supports the hypothesis that the declines in small mammals across northern Australia are driven by cat predation facilitated by simplification of ground layer structure. However, this evidence is not direct, and other possible mechanisms including trophic alterations [25] and disease [19] may still have a role. Further research needs to measure whether mortality of prey is greater in more open areas. Regardless, the magnitude of the impacts of cats globally [58] suggests that our findings provide a general mechanism for prey decline in ecosystems with grass-dominated understoreys.

The results presented here suggest that manipulation of habitat through careful management of fire and grazing could be used to reduce impacts of cats over large landscapes. Reducing the frequency of intense fires and removing introduced herbivores is likely to be beneficial for small mammals - especially if this management is focussed on naturally mammal-rich habitats, and if it increases ground cover. Vegetation structure is pivotal in creating ideal landscapes for predators to hunt, and/or refuges for prey to hide.

## Supporting Information

Material S1 Details on the creation of the dynamic grass cover map.
(DOCX)
