# Supplementary material for: Landscape Management of Fire and Grazing Regimes Alters the Fine-Scale Habitat Utilisation by Feral Cats
Source: PLoS One. 2014 Oct 15;9(10):e109097. doi: 10.1371/journal.pone.0109097 (PMC4198095; doi:10.1371/journal.pone.0109097)
Supplement: Material S1 — Details on the creation of the dynamic grass cover map. (DOCX) [file pone.0109097.s001.docx]

**Supplementary material**

**Material S1.**

**Details on the creation of the dynamic grass cover map**

To accurately measure grass variables in a landscape of constantly changing grass biomass, we developed a dynamic map that estimated grass cover at any given location and date. This involved creating a series of models of grass cover, derived from vegetation attributes measured at 768 plots. These plots were each 10 m^2^, and spread across the study area and duration. To encompass a full range of fire and grazing responses in grass growth, we used a stratified random sampling design with six plots placed in each grass community (see Table 1) and to each combination of mild / intense fire; in four time periods (up to 1 month post fire, 2 – 12 month post fire, 12 – 24 month post fire, greater than 25 months since fire; in grazed and destocked areas. This created a total of 96 plots per community.

At each of the 768 plots, we estimated the extent of grass cover at different heights by adapting a line-intercept method. First, dominant plant species were identified. Then, we inserted a 100 cm pole (diameter of 1.5cm) vertically through the grass to the ground at 50 points in a systematic grid over the plot. The number of grass intercepts were recorded in height brackets of 0 – 10 cm (cat paw height), 11 – 30 cm (cat body height), and 31 – 100 cm (greater than cat body height). For each plot, we converted this into grass cover (% of poles with any grass intercept higher than 10 cm), dense grass cover (% of poles with 10 or more grass intercepts; typically the centre of tussocks), and grass biomass (total sum of grass intercepts). We considered grass cover relevant for cats to be that higher than their paws (~ 10 cm), and grass cover less than this would not assist their concealment or impair their vision. Therefore, cover was measured as the percent of poles with any grass intercept greater than 10cm.

To determine values for grass variable (cover, dense cover and biomass) for any given location and date in the study area, models were derived from values for each plot and plotted against other spatial and temporal explanatory variables that we could measure or map. These were grass community class (as Table 1, below), time since fire (mapped as per methods in manuscript), estimated number of days since fire where grass had water available to grow (one month after last rain > 5ml at Mornington), number of days since the start of the dry season (days after April 1^st^, until December 1^st^), intensity of fire (high intensity versus mild), and grazing by introduced herbivores (delineated by the Australian Wildlife Conservancy’s’ destocking fence). For each grass community, we fitted a linear model to every combination of time since fire, fire intensity, dry days, grazing, and an interaction term of time since fire and grazing. Within each grass community, these models were compared within an information theory framework, and the most parsimonious model was determined as that with the lowest AICc and Aikike weight. All analysis was conducted in R version 3.2 using the ‘nlme’ and ‘MuMIn’ packages.

Once the most parsimonious model was selected for each grass community, we applied the parameter estimates to each cat fix and associated random fix based on values of predictor variables. We also capped derived values at an upper and lower limit; zero was defined as the lower limit; the upper limit was the average value for the corresponding unburnt plots in each grass communities and grazing type. This allowed us to predict the grass cover, dense grass cover and relative grass biomass at any location within the study area given grass community, time since fire and stocking treatment. All top models had an adjusted R^2^ of greater than 0.7.

Table 1. Parameter estimates used to derive grass cover (%), based on linear models fitted to field measurements for each grass community. ‘Growdays’ is number of days since fire when grass could grow (one month post rain). Grazing is whether site was stocked with large feral herbivores. ‘Dry months’ is number of months into dry season (starting April, ending December). ‘Maximum value’ is the upper limit where values were capped, based on the average grass cover for unburnt plots.

|  | Parameter estimates for each grass community | | | | | |  | Maximum value | |
| --- | --- | --- | --- | --- | --- | --- | --- | --- | --- |
| Grass community | intercept | growdays (sqrt) | growdays (sqrt)  × grazing? | grazing | dry months | Early fire |  | Stocked | Destocked |
| Riparian forest | 8.23 | 3.78 | 0 | -30 | 0 | 15 |  | 31 | 60 |
| Alluvial grasslands | -0.68 | 5.76 | 0 | -26.35 | -6.29 | 4 |  | 75 | 92 |
| Bluegrass plains | 2.49 | 4.77 | 0 | -29.66 | 0 | 5 |  | 74 | 90 |
| Canegrass | 5 | 3.79 | 0 | 0 | 0 | 0 |  | 96 | 89 |
| Mixed woodlands | 7.07 | 5.08 | 0 | -19.11 | -3.37 | 3 |  | 72 | 91 |
| Sandseep | -17.25 | 6.93 | 0 | -10.9 | -4.53 | 7 |  | 98 | 96 |
| Hillside woodlands | 11.58 | 3.69 | 0 | 0 | -15.1 | 2 |  | 80 | 91 |
| Spinifex woodlands | -0.92 | 3.75 | -0.761 | 2.54 | -2.14 | 5 |  | 70 | 70 |
| Bare ground | 0 | 0 | 0 | 0 | 0 | 0 |  |  |  |
